# Insulin-related decrease in cerebral glucose despite normoglycemia in aneurysmal subarachnoid hemorrhage

**DOI:** 10.1186/cc6776

**Published:** 2008-01-24

**Authors:** Florian Schlenk, Daniela Graetz, Alexandra Nagel, Maren Schmidt, Asita S Sarrafzadeh

**Affiliations:** 1 Department of Neurosurgery, Charité Campus Virchow Medical Center, Augustenburger Platz, 13353 Berlin, Germany; 2 Department of Anaesthesiology and Intensive Care Medicine, Charité Campus Virchow Medical Center, Augustenburger Platz, 13353 Berlin, Germany

## Abstract

**Introduction:**

Hyperglycaemia following aneurysmal subarachnoid hemorrhage (SAH) is associated with complications and impaired neurological recovery. The aim of this study was to determine the effect of insulin treatment for glucose control on cerebral metabolism in SAH patients.

**Methods:**

This prospective, nonrandomized study was conducted in 31 SAH patients in an intensive care unit (age 52 ± 10 years, World Federation of Neurological Surgeons grade 2.9 ± 1.6). A microdialysis catheter was inserted into the vascular territory of the aneurysm after clipping. Blood glucose levels above 140 mg/dl were treated with intravenous insulin and the microdialysates were analyzed hourly for the first 12 hours of infusion.

**Results:**

No hypoglycaemia occurred. Twenty-four patients were treated with insulin for glucose control. Higher age and World Federation of Neurological Surgeons score were risk factors for need for insulin treatment (*P *< 0.05). Although blood glucose remained stable after initiation of insulin infusion, insulin induced a significant decrease in cerebral glucose at 3 hours after onset of the infusion until the end of the observation period (*P *< 0.05), reflecting high glucose utilization. The lactate:pyruvate ratio and glutamate did not increase, excluding ischaemia as possible cause of the decrease in glucose. Glycerol tended toward higher values at the end of the observation period (9 to 12 hours), reflecting either tissue damage after SAH or the beginning of cellular distress after insulin infusion.

**Conclusion:**

Higher SAH grade was among the risk factors for need for insulin. Intensive glycaemic control using insulin induced a decrease of cerebral glucose and a slight increase in glycerol, though blood glucose remained normal. Future studies might detect relevant metabolic derangements when insulin treatment starts at low cerebral glucose levels, and may allow us to design a strategy for avoidance of insulin-induced metabolic crisis in SAH patients.

## Introduction

Hyperglycaemia on admission and elevated blood glucose levels during the first week after SAH are common and well established as predictors of poor outcome [1,2]. Although preventative glycaemic control with levels not substantially exceeding 110 mg/dl reduced mortality during intensive care by more than 40%, threshold glucose levels deemed to require treatment with insulin in patients with aneurysmal subarachnoid haemorrhage (SAH) vary considerably [1-3].

One of the main advantages of cerebral microdialysis (an established neuromonitoring technique for analyzing cerebral extracellular fluid) is its ability to assess cerebral delivery and utilization of glucose, the main source of energy to the brain, online in the neurointensive care unit [4-6]. Data from patients with traumatic brain injury (TBI) [7] demonstrated a clear relationship between low cerebral microdialysis glucose levels and unfavourable outcome. Similarly, in SAH lowered cerebral glucose levels were accompanied by a severe metabolic derangement [8]. Furthermore, targeted insulin therapy for glucose control was shown to reduce cerebral extracellular glucose and to increase markers of cellular distress in TBI [9]. Although treatment of hyperglycaemia improves outcome, insulin might have harmful effects by inducing hypoglycaemia and a metabolic crisis caused by low cerebral glucose levels. Lacking the data to address this issue in SAH patients, we conducted the present study to investigate the potentially harmful effect of insulin treatment for blood glucose control on cerebral metabolism in patients following aneurysmal SAH.

## Materials and methods

### Patient population

This study was approved by the local research ethics committee at Charité Virchow Medical Center, in accordance with the Declaration of Helsinki as revised in Edinburgh in October 2000. Written informed consent was obtained from each patient or their closest family relative.

### Patient characteristics and management

This study forms part of an ongoing prospective study on cerebral metabolism monitored by bedside microdialysis in aneurysmal SAH patients. A total of 31 patients were enrolled, who were admitted to the neurosurgical department of a university hospital between August 2005 and June 2007 and who met the following criteria: confirmation of SAH by head computed tomography; presence of intracranial aneurysm(s), as demonstrated by cerebral angiogram; and surgical therapy. All patients considered surgical candidates were managed in accordance with a uniform protocol detailed previously [5]. Depending on their neurological course, the patients were classified as asymptomatic (*n *= 6) or as exhibiting symptoms of acute focal deficit (AFND; *n *= 14) or delayed ischaemic neurological deficit (DIND; *n *= 11), with detailed definitions described previously [5]. Information compiled for each patient included the following: haemodynamic parameters, respiratory values, laboratory test results, fluid balance data and chest radiograph findings. Glucose levels were targeted to be under 140 mg/dl using intravenous insulin if necessary. Routine glycaemic control consisted of blood glucose checks every 4 hours (6 hours in patients without insulin treatment) and goal-directed adjustment of insulin infusion by the bedside nurse.

### Bedside microdialysis

A microdialysis catheter (CMA 70; CMA Microdialysis, Solna, Sweden; length 10 mm, molecular weight limit of 100 kDa) was inserted immediately after aneurysm clipping into brain parenchyma of the respective vascular territory of the aneurysm (for instance, the right frontal lobe in patients with an anterior communicating artery aneurysm). Care was taken to avoid insertion into macroscopically lesioned brain tissue or into an intracerebral haemorrhage. Catheters were perfused with sterile Ringer's solution at a flow rate of 0.3 μl/min. On the outlet tube, perfusates were collected in microvials, exchanged hourly and analyzed immediately at bedside in a mobile photometric, enzyme-kinetic analyzer (CMA 600; CMA Microdialysis). The estimated recovery for the system is 0.65 to 0.72 [10]. Parameters of energy metabolism (glucose, pyruvate, lactate, lactate:pyruvate ratio), glycerol (a marker of cell membrane degradation) and glutamate (a marker of ischaemia) were analyzed [6,11,12]. In a series of studies, changes in the lactate:pyruvate ratio and glutamate were shown to indicate early the onset of delayed neurological deterioration and to be in good accordance with the clinical course of SAH patients [4,5,11-15]. The lactate:pyruvate ratio was the best metabolic prognostic marker of 12-month outcome in SAH [16].

Microdialysis data are presented as microdialysate concentrations. The occurrence of critical decreases in cerebral glucose (defined as decrease to <0.6 mmol/l, corresponding to the mean level minus 1 standard deviation of our asymptomatic SAH patients [8]) was recorded.

### Data analysis

All data were collected during days 1 to 10 after admission for SAH. Between-group comparisons were performed using Kruskal-Wallis one-way analysis of variance. Statistical analysis of non-normally distributed sequential data over time was performed using Wilcoxon signed-rank test. Correlations between normally distributed interval-scaled data were calculated using Pearson's product-moment correlation coefficient. Otherwise, Spearman's rank correlation coefficient was calculated. Data in the tables and text are expressed as mean ± standard deviation if not specified otherwise. Differences were considered statistically significant at *P *< 0.05. All statistical analyses were conducted using SPSS 14.0 (SPSS Inc., Chicago, IL, USA).

## Results

### Patient characteristics

Demographic and clinical characteristics of the 31 patients are summarized in Table 1. Patients were classified as an insulin treatment group (*n *= 24) and a noninsulin treatment group (*n *= 7). All patients (*n *= 31) underwent early surgery (within 72 hours after initial bleeding). Age, admission World Federation of Neurological Surgeons (WFNS) score and Fisher score were significantly higher in the insulin treatment group (Table 1).

**Table 1:** Patient characteristics

Characteristic	Treatment group		P
		
	Insulin treatment (*n *= 24)	No insulin treatment (*n *= 7)	
Age (years)	54.0 ± 9.5	46.0 ± 8.7	*0.04*
Sex (male/female; *n*)	7/17	1/6	*0.44*
Clinical presentation (asymptomatic/AFND/DIND; *n*)	1/14/9	5/0/2	*0.03*
Admission WFNS score	3.3 ± 1.6	1.6 ± 0.8	*0.02*
I (*n *[%])	5 (21)	4 (57)	
II (*n *[%])	4 (17)	2 (29)	
III (*n *[%])	2 (8)	1 (14)	
IV (*n *[%])	6 (25)	0 (0)	
V (*n *[%])	7 (29)	0 (0)	
Fisher score	3.7 ± 0.6	3.2 ± 0.8	*0.06*
Time between SAH and surgery (hours)	14.5 ± 7.8	15.4 ± 7.9	*0.72*
Duration of microdialysis (hours)	192.6 ± 71.0	295.1 ± 66.4	*0.87*
*Intensive care unit stay (days)*	16.8 ± 10.4	*12.8 ± 7.8*	*0.40*

Characteristics of 31 subarachnoid haemorrhage (SAH) patients. Data are expressed as mean ± standard deviation or absolute number and percentage; *P *values are given for between-group comparison of insulin-treated and noninsulin-treated patients (Kruskal-Wallis one-way analysis of variance). WFNS, World Federation of Neurological Surgeons Grading of SAH [21]; AFND, acute focal neurological deficit; DIND, delayed ischemic neurological deficit.

### Blood glucose and insulin treatment

In 92% of the insulin-treated patients, hyperglycaemia was present on admission (86% in the noninsulin treatment group) and in all patients on at least one of the following 10 days in the intensive care unit. Continuous intravenous insulin treatment started 2.6 ± 3.0 days after admission. The median blood glucose at onset of insulin infusion was 142.0 ± 7.6 mg/dl. No episodes of insulin-induced hypoglycaemia (lowest value 80 mg/dl) occurred during 10 days after SAH. A critical decrease in cerebral glucose (to <0.6 mmol/l) occurred in 19 patients (79%) of the insulin treatment group, beginning 99.1 ± 54.4 hours after initiation of insulin infusion (duration 6.9 ± 11.6 hours) and in three patients in the noninsulin treatment group. In older patients (*P *= 0.03) and males (*P *= 0.02), low cerebral glucose values during insulin infusion occurred more frequently than in younger patients and females. There was no difference in the incidence and duration of critically low cerebral glucose values (<0.6 mmol/l) between the insulin treatment and noninsulin treatment groups.

### Cerebral metabolic changes during insulin treatment

**Figure 1:**
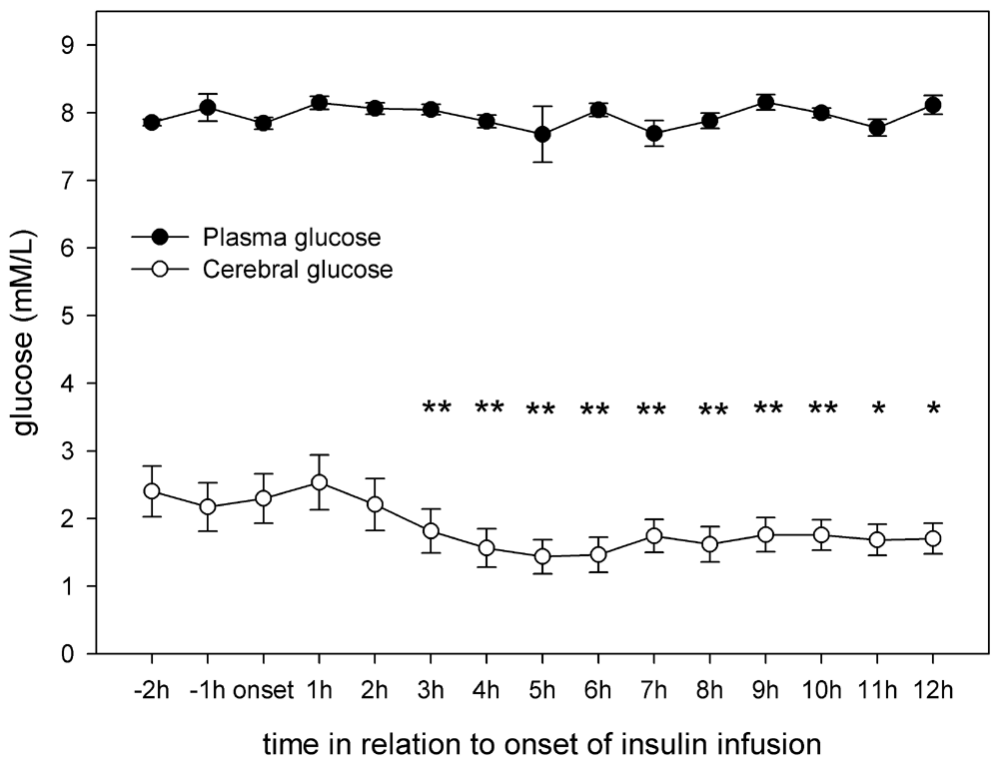
Course of blood and cerebral extracellular glucose during insulin infusion. Data are expressed as mean ± standard error of blood glucose and hourly microdialysate concentrations from 24 patients treated with continuous intravenous insulin. Blood glucose levels were not available for every single hour from every patient (see text for details). Levels of significance are indicated for comparison with microdialysate concentrations at the start of insulin infusion (Wilcoxon signed-rank test). **P < 0.01; *P < 0.05.

**Figure 2:**
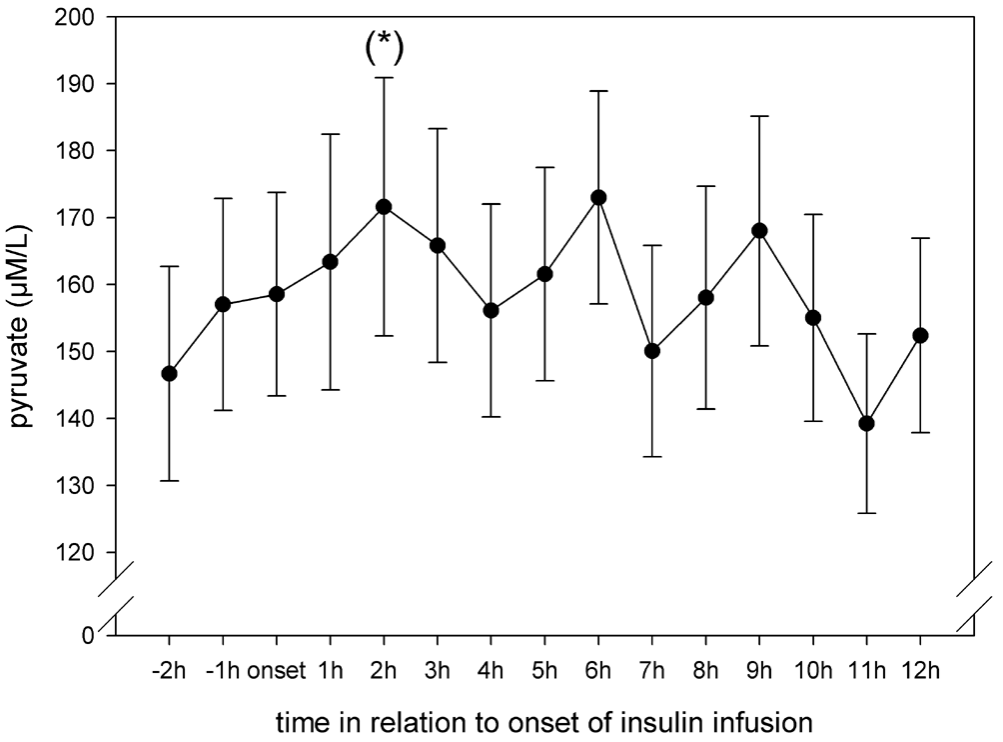
Course of cerebral extracellular pyruvate during insulin infusion. Data are expressed as mean ± standard error of hourly microdialysate concentrations from 24 patients treated with continuous intravenous insulin. Levels of significance are indicated for comparison with microdialysate concentrations at the start of insulin infusion (Wilcoxon signed-rank test). (*)*P *< 0.1.

**Figure 3:**
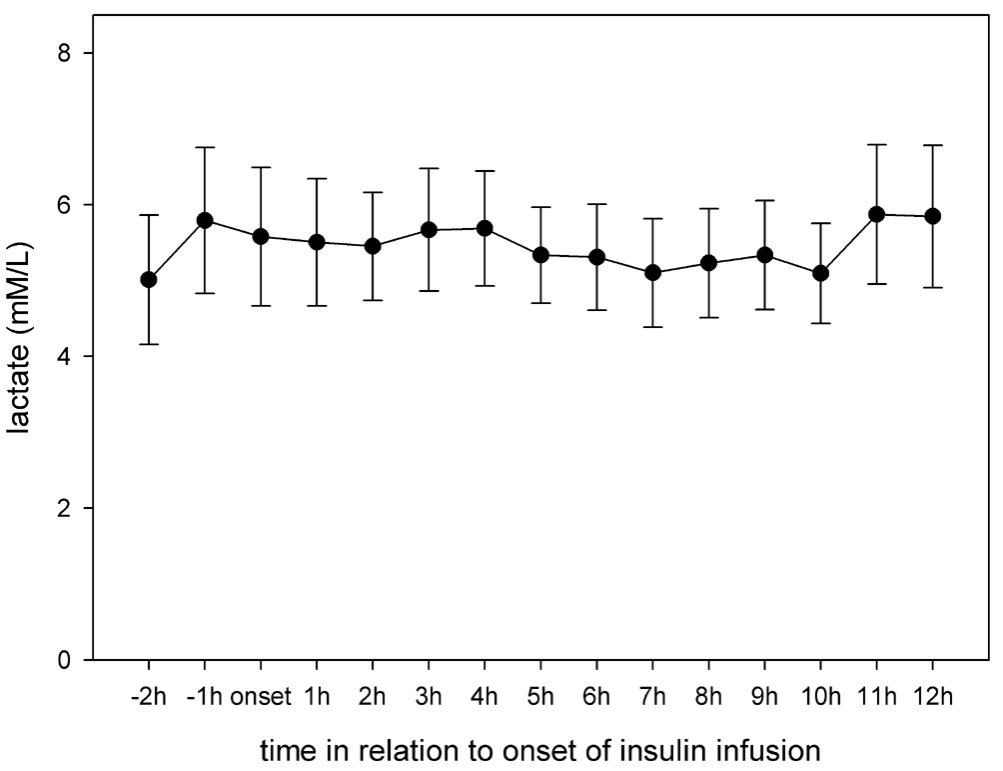
Course of cerebral extracellular lactate during insulin infusion. Data are expressed as mean ± standard error of hourly microdialysate concentrations from 24 patients treated with continuous intravenous insulin.

**Figure 4:**
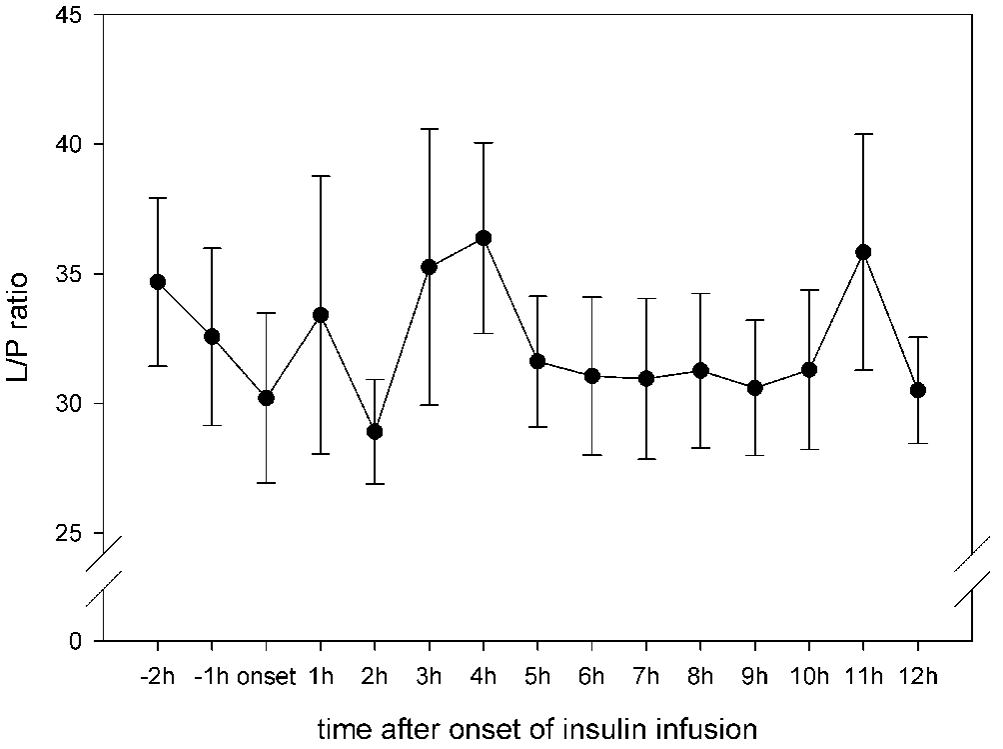
Course of cerebral lactate/pyruvate ratio during insulin infusion. Data are expressed as mean ± standard error of hourly microdialysate levels from 24 patients treated with continuous intravenous insulin. L/P, lactate:pyruvate.

**Figure 5:**
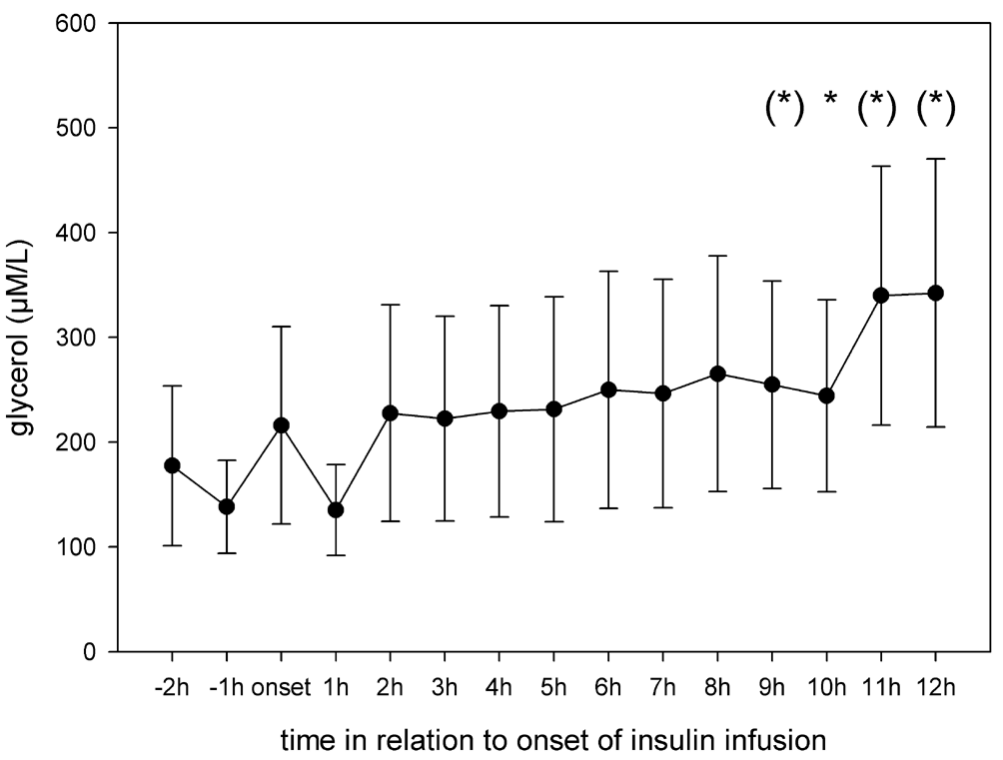
Course of cerebral extracellular glycerol during insulin infusion. Data are expressed as mean ± standard error of hourly microdialysate concentrations from 24 patients treated with continuous intravenous insulin. Levels of significance are indicated for comparison with microdialysate concentrations at the start of insulin infusion (Wilcoxon signed-rank test). *P < 0.05; (*)*P *< 0.1.

**Figure 6:**
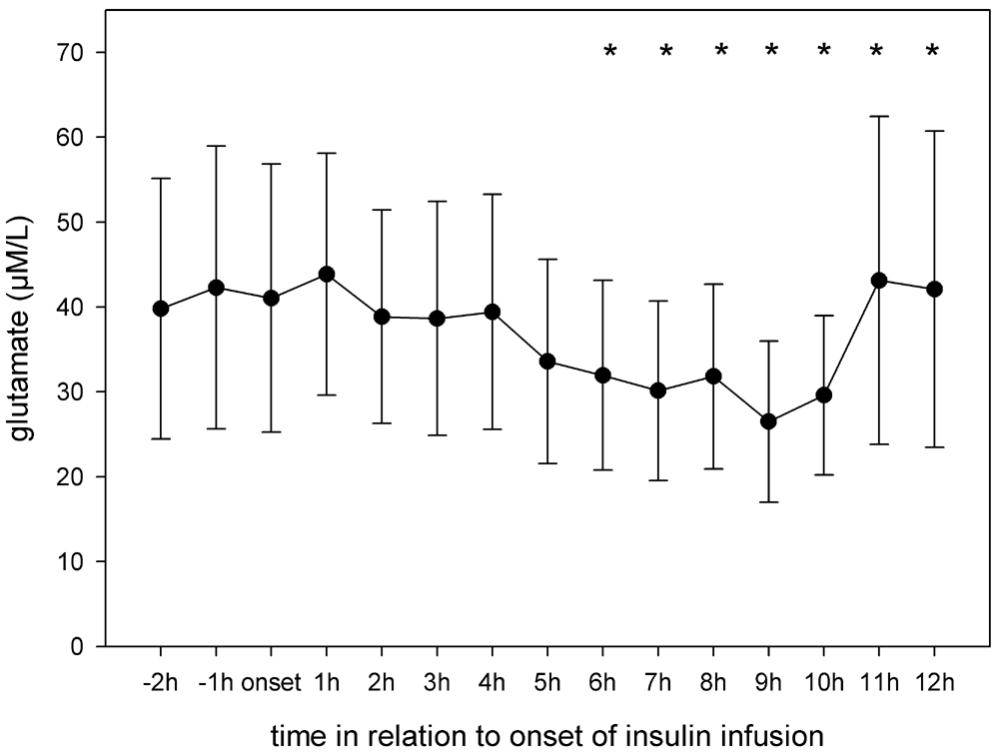
Course of cerebral extracellular glutamate during insulin infusion. Data are expressed as mean ± standard error of hourly microdialysate concentrations from 24 patients treated with continuous intravenous insulin. Levels of significance are indicated for comparison with microdialysate concentrations at onset of insulin infusion (Wilcoxon signed-rank test). *P < 0.05

Results from Vespa and colleagues [9] in patients with TBI showed that microdialysis glucose reached its nadir at 11 ± 1 hours after onset of insulin infusion. Hence, in the present study the microdialysis parameters were analyzed hourly for 12 hours after initiation of insulin infusion. Interestingly, although blood glucose remained stable after insulin onset, cerebral glucose began to decrease significantly at 3 hours after the start of infusion and remained low until the end of our observation period (Figure 1). Extracellular values of pyruvate, lactate and lactate:pyruvate ratio did not change during insulin infusion (Figures 2 to 4). Glycerol, a marker of cellular membrane degradation, tended toward higher values at the end of the observation period (9 to 12 hours; Figure 5). This might either reflect tissue damage after SAH or indicate cellular distress following insulin-induced decrease in cerebral glucose. Extracellular concentrations of glutamate decreased during insulin infusion (hours 6 to 12; *P *< 0.05), indicating that no additional ischaemia was present during the observation period (Figure 6). Furthermore, the decrease in glutamate might reflect metabolic improvement caused by treatment of hyperglycaemia.

### Cerebral metabolic changes and clinical symptoms in relation to insulin

It must be considered that a decrease in cerebral glucose might not be caused by insulin treatment alone but can also reflect an increased consumption or decreased delivery caused by cerebral vasospasm. To evaluate whether the occurrence of vasospasm led to reductions in glucose that were independent of the use of insulin, we analyzed our data according to the onset of DIND.

**Figure 7:**
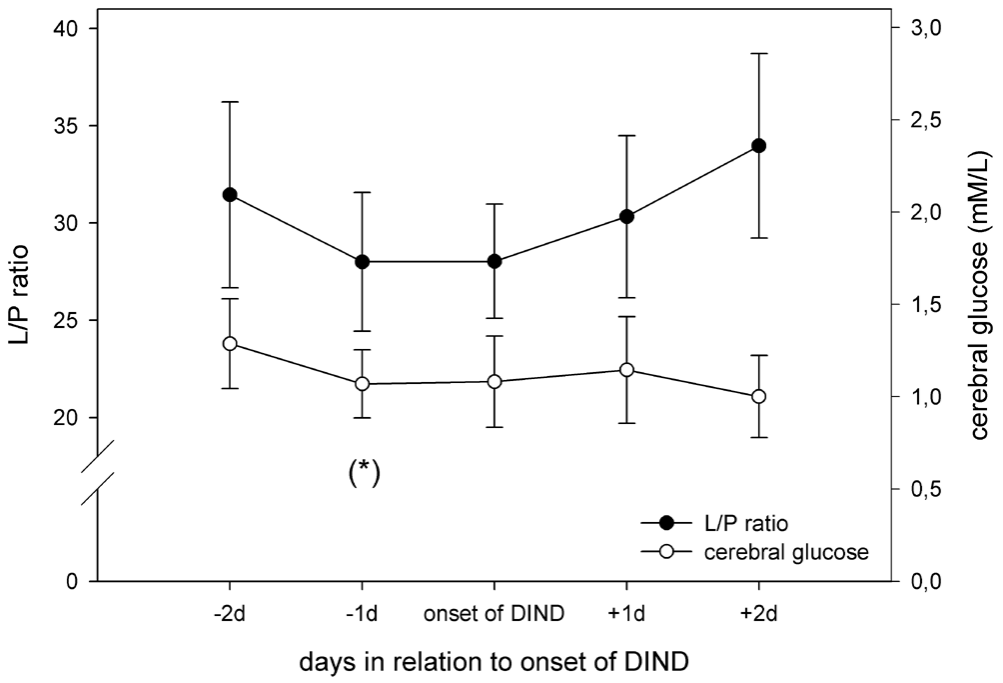
Course of cerebral extracellular glucose and lactate/pyruvate ratio in insulin-treated SAH patients developing DIND. Data are expressed as mean ± standard error of daily medians calculated from hourly measured microdialysate levels of glucose and lactate:pyruvate (L/P) ratio in nine insulin-treated subarachnoid haemorrhage (SAH) patients developing delayed ischaemic neurological deficit (DIND). (*) indicates a trend (*P *< 0.1) toward a decrease in cerebral glucose, comparing the levels at 2 days and 1 day before onset of DIND (Wilcoxon signed-rank test, *P *= 0.068).

Eighty-two per cent of the DIND patients were treated with insulin infusion (9/11), and in almost all of them (8/9) the insulin infusion was started before the onset of DIND. Cerebral glucose tended to decrease 1 day before clinical manifestation of DIND (*P *= 0.068; Figure 7), suggesting that the decrease in glucose may not be caused by DIND but possibly by insulin infusion, which started in average 2 days before DIND. The lactate:pyruvate ratio and glycerol level (not shown) did not change during this short period of time; however, this is not entirely unexpected (Figure 7).

## Discussion

The high incidence of hyperglycaemia identified in this study confirms the need to study the effect of insulin in detail. In 77% of the patients included in the study, insulin was necessary for glycaemic control. There are two principal findings of this study. First, older age and higher WFNS and Fisher scores were risk factors for need for insulin treatment. Second, although blood glucose remained stable during the first 12 hours of insulin infusion, cerebral glucose began to decrease significantly at 3 hours after the start of infusion and remained low until the end of the 12-hour observation period. The lactate:pyruvate ratio and glutamate level did not increase, excluding ischaemia as a possible cause of the decrease in glucose. After 8 hours of insulin infusion, glycerol (a marker of cell membrane degradation) increased, reflecting either tissue damage after SAH or the beginning of cellular distress following insulin-induced decrease in cerebral glucose.

The role of insulin in the metabolic processes of the brain remains unclear, but recent studies in the human brain using 2-deoxy-2-[^18^F]fluoro-D-glucose (FDG) positron emission tomography (PET) [17] indicated that despite an abundance of insulin receptors in the brain tissue, brain glucose metabolism is not sensitive to insulin in physiological concentrations. Unexpectedly, in this study intracerebral glucose concentrations decreased after insulin, although blood glucose levels remained unaffected. This observation is supported by data from TBI patients showing that intensive insulin therapy results in a net reduction in microdialysis glucose and an increase in both markers of cellular distress and oxygen extraction fraction to near ischaemic levels, even in absence of profound hypoglycaemia [9].

In our study, mean cerebral glucose concentrations did not fall below a critical threshold during insulin infusion. However, the marked decrease in cerebral glucose that could be observed suggests that in patients already presenting low cerebral glucose levels before onset of insulin infusion, cerebral glucose concentrations could decrease to critically low levels and compromise the cerebral energy metabolism substantially. Therefore, in our view, monitoring of cerebral glucose, especially at the start of and during insulin treatment, is valuable. This is even more the case in high-grade SAH patients as insulin treatment mainly becomes necessary; these patients are known to develop a deranged cerebral metabolism and unfavourable outcome. Although metabolic disturbances (increase in glycerol) could be observed even at normal blood and cerebral glucose levels, future studies might detect a relevant metabolic derangement when insulin is administered to patients with cerebral glucose levels that are already critically low. This might be relevant because it has been shown that low cerebral glucose levels (<0.2 mmol/l) correlate with unfavourable outcome in TBI patients [7].

### Hyperglycaemia and insulin treatment

Hyperglycaemia is common in critically ill patients. After stroke, the in-hospital mortality was shown to be lowest in normoglycaemic patients (1.7%), twice as high in diabetes patients (3%) and increased by a factor of 10 in patients with stress-induced hyperglycaemia (16%) [18]. In the latter group, insulin treatment became necessary thrice as frequently. Because hyperglycaemia appears to be injurious in conditions of brain ischaemia, the findings of others and our own group suggest that avoidance of hyperglycaemia should be a general strategy in SAH patients [1,2,16,19,20]. However, until now it was unclear whether this strategy could be endorsed for application in patients with low extracellular glucose levels. Data from TBI patients support the concern that a reduction in serum glucose could create substrate limitation in the injured brain [9]. In the present study, during continuous insulin infusion, there was a decrease in cerebral glucose levels in 79% of patients, similar to the percentage previously observed in TBI patients (79%) [9]. This decrease in cerebral glucose was associated with deterioration in homeostasis of cerebral metabolism in TBI patients.

### Critically lowered cerebral glucose

No episodes of insulin-induced hypoglycaemia (lowest value 80 mg/dl) occurred during 10 days after SAH. It is difficult to determine what level of glucose might be too low for adequate brain function, especially because in these patients the demand for energy may be increased regionally. Dynamic PET scanning of labelled water (H_2_^15^O) and deoxyglucose (^18^FDG) has revealed regional differences in cerebral metabolic capacity that may explain why cerebral cortex is more sensitive to an impaired glucose metabolism than other brain regions such as cerebellum [17]. It was shown that low cerebral glucose levels (<0.2 mmol/l) correlate with unfavourable outcome in TBI patients [7], which is supported by our clinical experience; hence, the suggested levels of 0.6 mmol/l might serve as an applicable threshold for a critical decrease in cerebral glucose [8]. In most patients (insulin treated: 83%; noninsulin treated: 77%) critically low cerebral glucose values were identified, but they occurred as late as 4.1 ± 2.3 days after the start of insulin infusion. The long time interval (>4 days) and the lack of any difference in their incidence between insulin-treated and noninsulin-treated patients suggest that these low cerebral glucose levels were unrelated to insulin treatment.

### Limitations of this study

There are some limitations of the present study. Microdialysis is a regional method, and the volume of brain tissue monitored by the microdialysis catheter covers only a few millimeters from the membrane. Incomplete recovery affects the measured absolute concentrations and impairs the comparison with blood data [10]. Additionally, because blood glucose values were monitored discontinuously and not hourly, like the microdialysates, some effects of insulin on blood glucose might not have been detected in these patients.

Our results support the view of Vespa and colleagues [9] that forced normalization of elevated blood glucose using insulin might be deleterious for brain glucose concentrations and provoke further metabolic crisis. This, however, can only be evaluated in future studies; further research, ideally in combination with PET or cerebral blood flow studies, might investigate whether a normalization of critically low microdialysis glucose levels can improve outcome in patients with cerebral lesions.

## Conclusion

This study confirms that hyperglycaemia is a significant complication in aneurysmal SAH. Higher SAH grade, Fisher score and age were risk factors for need for insulin. Insulin treatment for glycaemic control was safe with respect to blood glucose, because no hypoglycaemia (<80 mg/dl) occurred. However, insulin infusion induced a significant decrease in cerebral glucose and an associated increase in glycerol, reflecting high glucose utilization and possibly the beginning of cellular distress. Because slight metabolic disturbances could be observed even at normal cerebral glucose levels, future studies might detect a relevant metabolic derangement when insulin treatment starts at cerebral glucose levels that are critically low (<0.6 mmol/l). This could be relevant because it was shown that low cerebral glucose (<0.2 mmol/l) is associated with unfavourable outcome in TBI patients [7]. Future, more detailed studies are necessary to design optimized targeted therapies to avoid an insulin-induced metabolic crisis in SAH patients.

## Key messages

• Older age and higher WFNS and Fisher scores are risk factors for need for insulin treatment in patients with aneurysmal SAH.

• Insulin infusion appears to induce a decrease in cerebral extracellular glucose, even when blood glucose remains within the normal range.

• Monitoring of cerebral metabolism during insulin treatment in SAH is valuable because it allows one to detect metabolic derangements that may occur, especially when insulin treatment starts at low cerebral glucose levels.

## Abbreviations

DIND = delayed ischaemic neurological deficit; FDG = 2-deoxy-2-[^18^F]fluoro-D-glucose; PET = positron emission tomography; SAH = subarachnoid haemorrhage; TBI = traumatic brain injury; WFNS = World Federation of Neurological Surgeons.

## Competing interests

The authors declare that they have no competing interests.

## Authors' contributions

FS participated in the design of the study and performed the statistical analysis, created the tables and figures, and drafted part of the manuscript. He also managed the microdialysis monitoring and collected the microdialysis and blood samples of patients 1 to 15. DG and AN managed the microdialysis monitoring and ensured compliance with the study protocol in the intensive care unit, collected the microdialysis and blood samples of patients 16 to 31, and prepared the data for statistical analysis. MS performed the neurointensive management of the patients and guided the insulin treatment. AS conceived of the study and, as the project leader, was responsible for the design, coordination and data interpretation, and drafted part of the manuscript.
